# Antimicrobial Resistance Genes in *Legionella* from Artificial Water Systems: Findings from a Two-Year Study

**DOI:** 10.3390/antibiotics13121121

**Published:** 2024-11-23

**Authors:** Bernardo Beirão Pereira, Mário Marrafa, Carolina Cruz, Lúcia Rodrigues, Filipa Nunes, Silvia Monteiro, Ricardo Santos, Rui Neves Carneiro, Célia Neto, Joana Aguilar, Nuno Rafael Ferreiro, Margarida Passanha, Gonçalo Candeias, Aida Fernandes, Paulo Paixão, Maria Jesus Chasqueira

**Affiliations:** 1 Laboratory of Microbiology, Nova Medical School, Universidade Nova de Lisboa, 1169-056 Lisboa, Portugal; bernardo.pereira@nms.unl.pt (B.B.P.);; 2 Laboratório de Análises de Água, Instituto Superior Técnico, Universidade de Lisboa, 1049-001 Lisboa, Portugal; 3 Civil Engineering Reasearch and Innovation for Sustainability, Instituto Superior Técnico, Universidade de Lisboa, 1049-001 Lisboa, Portugal; 4 Departamento de Engenharia e Ciências Nucleares, Instituto Superior Técnico, Universidade de Lisboa, 1049-001 Lisboa, Portugal; 5 Direção de Laboratórios, Empresa Portuguesa das Águas Livres, 1250-144 Lisboa, Portugal; 6 Laboratório Regional de Saúde Pública, 4700-036 Braga, Portugal; 7 Laboratório Regional de Saúde Pública do Alentejo, 7000-811 Évora, Portugal; 8 Laboratório Regional de Saúde Pública Dra. Laura Ayres, 8135-014 Almancil, Portugal; 9 Comprehensive Health Reasearch Center, Nova Medical School, Universidade Nova de Lisboa, 1169-056 Lisboa, Portugal

**Keywords:** *Legionella*, *Legionella pneumophila*, antimicrobial resistance, ARG, LpeAB, Tet56

## Abstract

Background: *Legionella* species are the causative agent of Legionnaires’ disease and, as ubiquitous waterborne bacteria, are prone to antimicrobial resistance gene (ARG) acquisition and dissemination due to the antimicrobial contamination of natural environments. Given the potential health risks associated with ARGs, it is crucial to assess their presence in the *Legionella* population. Methods: The ARGs *lpeAB* and *tet56* were detected in 348 samples, isolates, and DNA extracts using conventional PCR. In a subset of *lpeAB*-positive isolates, azithromycin (AZT) MIC values were obtained using the EUCAST protocol and LpeAB activity was evaluated through an efflux pump inhibition assay. Results: The *lpeAB* gene was found in 19% (66/348) of samples, with higher detection rates in the *L. pneumophila* and *L. pneumophila* sg1 subgroups, at 30% and 41%, respectively. A positive association between *lpeAB* and *L. pneumophila* sg1 was found. The MIC values of the *lpeAB*-positive isolates ranged from 0.064 to 2 mg/L. LpeAB inhibition resulted in 2- and 4-fold MIC reductions in 10 of the 13 isolates analyzed. One sample each of *L. longbeacheae* and *L. bozemanae* was found to possess the *tet56* gene. Conclusions: The *lpeAB* gene is predominant in *L. pneumophila* sg1. A few isolates with the *lpeAB* gene exhibited MIC values below the EUCAST tentative highest MIC values for wild-type isolates. Expanding ARG monitoring in *Legionella* is essential to assess the public health risk of Legionnaires’ disease.

## 1. Introduction

*Legionella* is a waterborne Gram-negative bacterium capable of causing a self-limited flu-like Pontiac fever or a potentially fatal pneumonia known as Legionnaires’ disease (LD) [1] which corresponds to 4.6% of community-acquired pneumonia cases worldwide [2]. This bacterium is usually present in aquatic environments [3,4,5], whose aerosol dissemination capacity can increase public health risk.

Currently, we face significant challenges in the *Legionella* field spectrum. First, for the last decade, a continuous rise in the European average incidence of LD can be observed, from 1.13 cases per 100,000 inhabitants in 2013 to 3.23 in 2023. Portugal has accompanied this trend, increasing from 0.9 in 2013 to 3.45 in 2023, ranking within the top 10 notifying countries in the European Economic Area [6]. Second, an increase in loss of antimicrobial susceptibility has also been reported, mainly in *Legionella pneumophila* (Lp) strains, the most common species associated with human disease [7,8,9,10]. Loss of susceptibility can be related to antimicrobial exposure. This potential exposure of antimicrobials in water systems is derived from the contamination of agricultural and wastewater sources. Wastewater has previously been recognized as a common antimicrobial resistance gene (ARG) reservoir [9,10]. Furthermore, *Legionella* is capable of acquiring and disseminating genetic material through horizontal gene transfer (HGT) [11,12]. This phenomenon is a public health risk that must be controlled, as ARGs play a vital role in the loss of susceptibility to antimicrobials, which can lead to a decrease in treatment effectiveness.

Two ARGs have been described in *Legionella*: *lpeAB,* found predominantly in *Lp* serogroup 1 (*Lp* sg1) [13,14], which encodes a resistance nodulation division efflux pump homologous to *acrAB* in *E. coli*, and *tet56*, identified only in *Legionella* non-*pneumophila*, which encodes a tetracycline destructase homologous to TetX variants [15]. Since its recent description, the *lpeAB* gene has been frequently analyzed in environmental populations [8,14,16,17,18], while the same cannot be said for *tet56*. Nonetheless, including the *tet56* gene in broad searches is relevant, as HGT phenomena are common in *Legionella’s* ecological niche, especially in selective environments [19,20,21].

Given the fact that *Legionella* transmission occurs directly from the environment, with only one reported case of interhuman transmission [22], it is relevant to assess the presence of ARGs in *Legionella* that colonize artificial water systems and are capable of producing aerosols. The present study aimed to screen the presence of *lpeAB* and *tet56* genes in environmental *Legionella* populations collected over two years from aerosol-producing systems and equipment in Portugal. With this knowledge, it will be possible to foresee future problems and develop preventive measures that potentiate better risk management for public health.

## 2. Results

**Figure 1:**
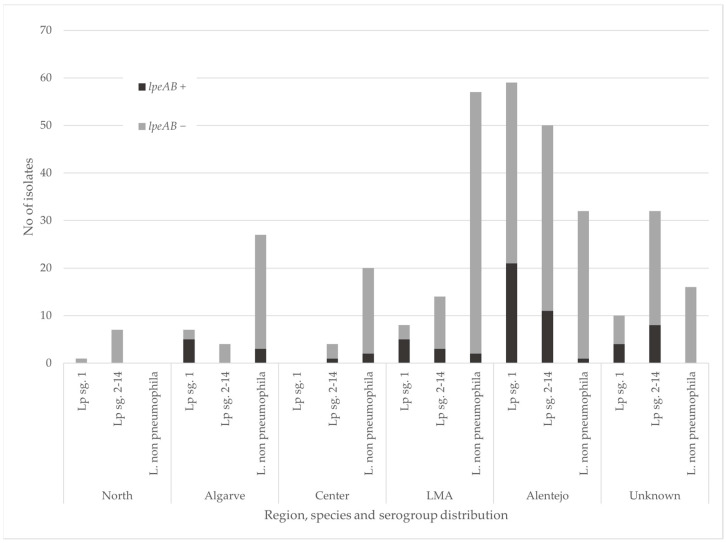
Sample distribution based on *lpeAB* detection, geographical origin, species, and *L. pneumophila* serogroups. LMA, Lisbon metropolitan area.

The 348 environmental samples analyzed were recovered from the water of different regions of Portugal and were collected over three years: 2021 (n = 23), 2022 (n = 173), and 2023 (n = 152), as described in Figure 1.

Overall, the majority of samples were from the regions of Alentejo (n = 141) and the Lisbon metropolitan area (LMA) (n = 79). Nationwide, *L. pneumophila* was the most frequent species detected (n = 196), with 111 being sg2-14 and 85 being sg1. However, *L*. non-*pneumophila* was predominant in LMA (n = 57), Algarve (n = 27), and the center (n = 20). Among the species identified were *L. anisa*, *L. longbeacheae*, and *L. dumoffii*. The vast majority of samples were obtained from building network waters (n = 322). Other sources included industrial waters (n = 16), superficial waters (n = 5), groundwater (n = 2), and wastewater (n = 1). Two were of unknown sources.

The *lpeAB* gene was found in 19% (66/348) of the samples, where the prevalence in *Lp* samples was significantly higher (*p*-value < 0.01) than in *L*. non-*pneumophila*—respectively 30% (58/196) and 5% (8/152). Interestingly, this gene was most frequent in *Lp* sg1 (41%, 35/85) (Figure 1). The statistical analysis revealed significant correlations between the presence of the efflux pump gene *lpeAB* and species/serogroup. The relationship between these variables is further explained with the odds ratio (OR), as the presence of *lpeAB* was positively associated with *Lp* sg1 (OR = 5.24). In contrast, a strong negative association was found between the presence of this gene with *Legionella* non-*pneumophila* (OR = 0.13).

All regions presented positive samples for *lpeAB*, apart from the north (n = 8), of which none presented the gene. Alentejo and Algarve displayed similar percentages of positive *lpeAB* samples—23% (33/141) and 21% (8/38), respectively. Positive rates for the center and LMA were 13% (3/24 and 10/79, respectively).

**Figure 2:**
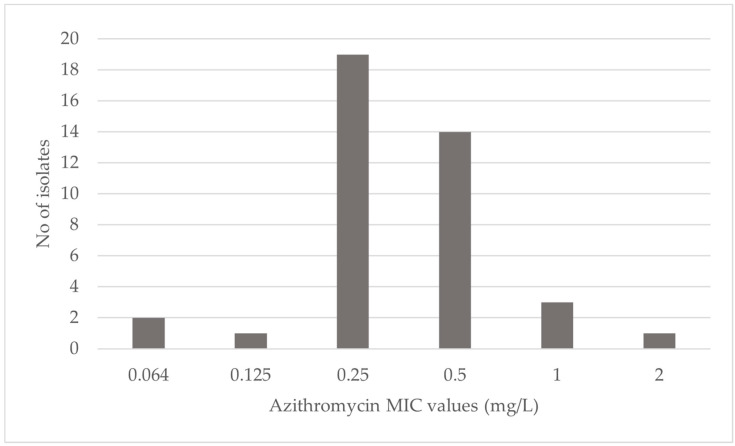
Azithromycin MIC distribution for *lpeAB-positive* isolates.

To assess the influence of the presence of *lpeAB* on the susceptibility to AZT, *lpeAB-positive* isolates (n = 40) were tested. The MIC distribution is described in Figure 2. Apart from three isolates, all were within the range described by EUCAST [23]. Overall, MIC values ranged from 0.064 to 2 mg/L, achieving MIC_50_ and MIC_90_ values of 0.25 and 0.5 mg/L, respectively.

The LpeAB inhibition assessment revealed a two-fold MIC reduction in 53% (7/13) of the isolates tested. One-third of the isolates presented a four-fold reduction, while two isolates did not show any alteration (Supplementary Materials).

Concerning the *tet56* gene, two positive samples were identified which had been previously classified as *Legionella* spp. Further identification with 16S sequencing for the extract and MALDI-TOF (VITEK MS V3, bioMérieux, Craponne, France) for the isolate revealed *L. bozemanae* and *L. longbeacheae,* respectively.

## 3. Materials and Methods

### 3.1. Bacterial Collection and DNA Extraction

Water samples positive for *Legionella* (n = 348), either via PCR or culture, were selected as the sample population in this study. Samples were collected between November 2021 and December 2023 and screened for the presence of *Legionella* following ISO/TS 12869:2019 and ISO 11731:2017 [24,25]. Samples were obtained in mainland Portugal from building network waters, industrial waters, superficial waters, groundwater, and wastewater.

Of the samples, 205 were DNA extracts and 143 were isolates, all of which were stored at −80 °C until gene examination. Isolates were preserved in a thioglycolate medium (Biokar Diagnostics, Beauvais, France) with 15% glycerol. All *Lp* DNA extracts supplied were initially screened via PCR using the *wzm* gene to identify those that were sg1. All *wzm*-negative samples were considered as *Lp* sg2-14.

DNA from the isolates was obtained using an InstaGene Matrix Kit (Bio-Rad, Hercules, CA, USA).

### 3.2. DNA Amplification and Detection

Serogroup classification for DNA extracts and ARG detection were performed using conventional PCR. Briefly, PCR was performed with 1x Master mix GoTaq**^®^** G2 Hot Start Taq Polymerase 2x (Promega, Madison, WI, USA) and the primers from Table 1 at 0.8 µM.

### 3.3. MIC Determination

Susceptibility to azithromycin (Merck, Darmstadt, Germany) was determined for *lpeAB*-positive isolates following EUCAST guidelines. Briefly, wells containing 40 μL of antibiotic solution, ranging from 0.016 to 4 mg/L following 2-fold dilutions, were inoculated with 160 μL of bacterial suspension to reach a final concentration of 1.2 × 10^7^ CFU/mL. Microplates were incubated for 48 h at 37 °C in a humidified atmosphere. Buffered yeast extract (BYE) media, prepared as previously described [27], and a bacterial suspension were used as negative and positive controls, respectively. All samples were assayed in duplicate.

### 3.4. Efflux Pump Inhibition Assay

The activity of LpeAB was assessed in 2023 *lpeAB*-positive isolates (n = 12) according to the protocol described by Cocuzza et al. [16]. Briefly, wells containing 50 μL of antibiotic solution ranging from 0.016 to 4 mg/L were inoculated with 50 μL of bacterial suspension and efflux pump inhibitor reserpine (Sigma Aldrich, MO, USA) was added to achieve a concentration of 0.125 mg/L. Plates were incubated for 48 h at 37 °C in a humidified atmosphere. BYE media and bacterial suspension were used as the negative and positive controls of bacterial growth, respectively. Additionally, an *lpeAB*-negative isolate was used as a control for the reserpine reaction.

### 3.5. Statistical Analysis

The Fisher–Freeman–Halton test was used with Monte Carlo simulation at a significance level of 99% to determine possible associations between variables. Statistical data analysis was conducted using SPSS version 29.0.0.0 (241) (IBM, Armonk, NY, USA).

## 4. Discussion

Antimicrobial resistance has been a growing concern since the first description of penicillin insensitivity in 1947. Since then, antimicrobial resistance has become rampant due to growing healthcare and agricultural overconsumption of antimicrobials [28]. The topic has gained international attention as it not only threatens human healthcare and food security but also compromises access to clean water and sanitation and has other subsequent socioeconomic implications [29].

Recently, several authors [7,9,10,14] have demonstrated the presence of resistance mechanisms against the two predominant antimicrobial classes used in LD therapy. The loss of susceptibility of environmental *Legionella* poses a higher risk to infection treatment; therefore, monitoring the presence of *Legionella* ARGs in the environment is essential, not only to control resistance dissemination but to optimize empirical treatment of LD. In the present research, a disproportionate number of *Legionella* samples were collected in building water networks. One explanation for this phenomenon, previously reported by other authors [5,30], could be that water samples collected from cooling systems and refrigeration towers are under more restrictive guidelines.

The percentage of *lpeAB*-positive samples varies significantly throughout the literature, and screening is commonly conducted on strains with decreased susceptibility to azithromycin. Indeed, acquired resistance mechanisms are most often associated with loss of susceptibility. Nonetheless, the presence of *lpeAB* without alteration of MIC has also been reported [7,31]. As such, it is important to evaluate gene detection in both sensitive and resistant populations to best characterize its true impact on MIC and ECOFF (epidemiological cutoff value) distributions. Herein, we observed a 19% *lpeAB* presence rate in the sample population (Table 2), a similar value as described by Svetlicic et al., who also analyzed a wide range of *Legionella* species [30]. However, unlike Svetlicic et al., we observed the presence of the *lpeAB* gene in 5% (8/152) of *Legionella* non-*pneumophila*. Curiously, the *lpeAB* rate reported herein was considerably lower than that of the previous one described by our team (Table 2); such a difference is most likely due to the significant increase in the sample population.

The absence of a statistically significant relationship between the geographical distribution and *lpeAB* presence points to a possible equivalent distribution of this ARG in the continental Portuguese *Legionella* environmental population. Nonetheless, it is necessary to consider the uneven distribution of samples across geographical regions, mainly in the north and central regions, with the autonomous regions of Madeira and Azores excluded due to the limited sample supply.

Compared with the overall *L*. spp. in this study, the presence rate of the *lpeAB* gene showed a 10% increase when considering the *Lp* subpopulation and a 20% increase in *Lp* sg1, as shown in Table 2. A higher prevalence in *Lp* and *Lp* sg1 is expected since this efflux pump was first described in *Lp* and predominantly in *Lp* sg1 [14]. The predominance in *Lp* and *Lp* sg1 was further confirmed by analysis of the OR, as it showed a negative correlation between this ARG and *Legionella* non-*pneumophila* and a positive correlation with *Lp* sg1 isolates. Nonetheless, it is important to consider the scarce number of publications that describe *lpeAB* distribution, particularly in *Legionella* non-*pneumophila*. This reduced number could inevitably lead to sample bias.

Additionally, the results highlight a serious concern given the clinical relevance of *Lp* sg1, as it is the serogroup most commonly associated with cases of LD. These findings reveal an increased concern about the dissemination of ARGs in *Legionella*, a problem that we must keep under close surveillance and control.

Furthermore, the variation within sample populations described in the literature might further hinder data interpretation, as several works have mentioned ARG screening in only AZT-resistant subpopulations. To confirm that the use of AZT-resistant subpopulations does not incur a biased analysis of *lpeAB* presence, we analyzed the MIC values for AZT in our *lpeAB*-positive isolate subpopulation (n = 40). As shown in Figure 1, the majority of *lpeAB-*positive isolates presented MIC values above the tentative highest MIC for *lpeAB*-negative isolates (0.125 mg/L), which aligns with the EUCAST guidelines. Nonetheless, three *Lp* sg2-14 isolates presented MIC values considered to be a wild type, indicating that the gene’s presence might not always be translated into a loss of susceptibility. Indeed, ARG activity can be regulated through several mechanisms, from gene expression to post-transcriptional alterations. To rule out alternative sources of resistance loss and confirm the role of the LpeAB efflux pump, LpeAB activity was assayed in a subset of 13 positive isolates. Decreases of two- and four-fold were observed in the MIC values of LpeAB-inhibited isolates. Our results confirm that loss of susceptibility is due to the presence of this ARG. Nonetheless, the decrease observed is of lesser magnitude than previously described, as Cocuzza et al. obtained reductions of 16- and 32-fold. Regarding the two isolates that did not show a decrease in MIC, one presented an original MIC within the wild-type range, which is possibly due to the efflux pump’s lack of activity or expression. The second isolate presented a non-wild-type MIC, which demonstrates that loss of susceptibility is likely due to other resistance mechanisms.

Tetracyclines are not the first line of treatment for LD or the empirical treatment of pneumonia [33] and are usually considered alternative treatments to quinolones and macrolides. However, alternative treatments are the last resort and, as such, the last line of treatment for critical patients. In such scenarios, the potential loss of susceptibility may reduce treatment options and, ultimately, compromise effective therapy. The Tet56 tetracycline destructase is active against older tetracyclines; however, other members of the TetX family can inactivate newer tetracyclines, such as tigecycline. Herein, the *tet56* gene was found in two *L*. non-*pneumophila* samples, specifically in *L. longbeacheae* and *L. bozemanae*. *Legionella* non-*pneumophila* species are frequently overlooked due to their small contribution to LD cases in Europe and North America, despite being reported as the second most common causative agent of LD by the 2021 ECDC Annual Epidemiological Report of Legionnaires’ Disease [3]. Additionally, these species—*L. longbeacheae*, in particular—play a major role in Australia and New Zealand, accounting for the majority of LD cases in this region [34,35]. As such, the *tet56* gene might not be an active player in clinical loss of susceptibility, but it is still necessary to closely monitor its environmental distribution to control its dissemination to other *Legionella* species and potential alterations that might confer an increased health risk.

To achieve a more robust representation of Portuguese *Legionella*, we included DNA extracts in our samples. Despite adding significant value for ARG monitoring, composing 59% of the sample population, they do not allow for phenotypic assays, hindering data analysis.

In light of this growing antimicrobial crisis, the United Nations has launched the One Health approach, which recognizes the interconnections between human, animal, plant, and environmental health [36]. LD presents itself at the intersection of the values of One Health, as it demonstrates a strong connection between human and environmental health. As such, environmental monitoring is crucial for the database on *Legionella* resistance, which is necessary to provide support and robustness to future ECOFFs and breakpoints defined by EUCAST. Further research would benefit from expanding and standardizing the sample size for each parameter under evaluation and, additionally, broadening the ARG and other resistance determinants to identify and analyze the possibility of novel resistance mechanisms in *Legionella*.

## 5. Conclusions

Herein, we described the distribution of the *lpeAB* and *tet56* genes in a Portuguese environmental *Legionella* population that generally agrees with the data described in the literature. Our data support the EUCAST’s LpeAB cutoff for AZT MIC values. However, the presence of the *lpeAB* gene can be found in isolates with MIC values that are lower than the cutoff. Additionally, a case of an elevated MIC value without LpeAB activity was described; as such, other resistance mechanisms cannot be disregarded in a low-resistance isolate. The data presented highlight the need to monitor ARGs, given their key role in antimicrobial susceptibility loss. Ideally, this effort must be maintained and expanded to include phenotypic antimicrobial resistance tests to more accurately describe the impact of the presence of ARGs. These data contribute directly to the awareness of the risk associated with the increased presence of these resistance genes in the environment and the possible implications for LD treatment.

## Figures and Tables

**Table 1:** Primers used in this study.

Genes	Primers		Reference
*wzm*	Fw	5′-TTA CCG CTT GCT TTT ATG GA-3′	[26]
	Rv	5′-CCT ATC AAC GCT CTT GGA AA-3′	
*lpeAB*	Fw	5′-GTG ATG ATT GTC TTA TTG GTG CGA-3′	[14]
	Rv	5′-ATG GCG TTT AAG ATG ATG GTG ATT-3′	
*tet56*	Fw	5′-ATG TCT AAA AAT ATC AAA ATT CTC GTC-3′	[15]
	Rv	5′-CTA TGA TGA TTC ATA TTG AGG TAA GG-3′	

**Table 2:** *lpeAB* prevalence values in other studies.

Ref.	Sources	Population Tested (Species)	Number of Isolates in the Study	*lpeAB* Prevalence (%)
Svetlicic (2023) [30]	Env.	*L.* spp.	39	15
This study	Env.	*L.* spp.	358	19
Cruz (2023) [7]	Env.	*L.* spp.	57	47
VW-C (2017) [14]	Clin. + Env.	*Lp*	541	23
Svetlicic (2023) [30]	Env.	*Lp*	25	24
This study	Env.	*Lp*	196	30
Cruz (2023) [7]	Env.	*Lp*	43	45
Mercante (2018) [32]	Clin. + Env.	*Lp* ST1	502	99,8
Svetlicic (2023) [30]	Env.	*Lp* ST1	5	100
Minetti (2024) [31]	Clin. + Env.	*Lp* sg1	100	8
This study	Env.	*Lp* sg1	85	41
Cruz (2023) [7]	Env.	*Lp* sg1	10	60

Env., environmental; Clin, clinical.

## Data Availability

The original contributions presented in the study are included in the article/Supplementary Materials; further inquiries can be directed to the corresponding author/s.

## Supplementary Materials

The following supporting information can be downloaded at: https://www.mdpi.com/article/10.3390/antibiotics13121121/s1, Table S1: Sample Data.
